# Cloning of upstream region and cellulose synthase operon genes involved in bacterial cellulose biosynthesis by *Gluconacetobacter hansenii *ATCC23769

**DOI:** 10.1186/1753-6561-8-S4-P176

**Published:** 2014-10-01

**Authors:** Carolina Véspoli de Melo, Tatiana Souza-Moreira, Sandro Roberto Valentini, Cleslei Fernando Zanelli, Sidney José Lima Ribeiro

**Affiliations:** 1 School of Pharmaceutical Sciences, UNESP, Araraquara, SP, Brazil; 2 Chemistry Institute, UNESP, Araraquara, SP, Brazil

## Background

Cellulose is a homopolysaccharide composed of extracellular D-glucose monomers connected by glycosidic linkages in *β*-1,4 conformation which can be synthesized by a variety of living organisms, possessing numerous applications in food, pharmaceutical, medical area, etc.[1][2]. In 1990, cellulose synthase operon encoding four proteins required for bacterial cellulose (BC) biosynthesis by *Gluconacetobacter hansenii *was isolated. It was demonstrated that these genes *bcsA, bcsB, bcsC *and *bcsD *were, together, required for maximum production of celulose [3]. Later, researches identified other relevant regions for bacterial cellulose biosynthesis that occur upstream *(cmcax *and *ccpAx *genes*) *and downstream of the operon (*bglx *gene) [4]. In this study, we aimed to clone the most important genes related to the BC synthesis. With the purpose of cloning those genes, the genomic DNA of the mentioned microorganism was extracted and proceeded with the amplification of *cmcax, ccpAx *and cellulose synthase operon genes. Later, the genes were ligated to a cloning vector, transformed in *Escherichia coli *and the identity of the clones was confirmed by sequencing and comparing with the GenBank using Blastn tool. The cloning achievement of the operon and upstream genes from *G. hansenii *and their overexpression will enable studies on BC synthesis improvement.

## Methods

Extraction of bacterial genomic DNA was carried out using cell lysis under heating in the presence of sodium dodecyl sulfate (SDS) and proteinase K, followed by purification with a phenol:chloroform:isoamylic alcohol solution (PCI) and subsequent dialysis in TE buffer pH 8.0. The cloning of the genes related to the production of bacterial cellulose was carried out with amplification reactions (PCR) using specifically designed primers in the presence of Pfx Plantinum (Invitrogen) or Phusion (New England Biolabs) polymerases. The cloned genes were ligated to pTZ57R/T vector, transformed into ultracompetent *Escherichia coli *(DH10B) and sequencing was carried out using Big Dye ^® ^Terminator v3.1 Cycle Sequencing Kit and the Genetic Analyzer 3130 (Applied Biosystems). Identity was confirmed with Blastn tool (Basic Local Alignment Search Tool).

## Results

PCR reactions using *G. hansenii *genomic material and specific primers yield the fragments with the corresponding size of the upstream region (2.1 kb) and of cellulose synthase operon (9.0 kb). Finally, the sequencing of the cloned fragments allowed the confirmation of the identity of the genes *cmcax *and *ccpAx *and the genes of the BC operon from *G. hansenii*.

## Conclusions

The obtained clones showed high identity with the genes located in the upstream region of the cellulose synthase operon and with the genes in the BC operon. The next step will allow the overexpression of these genes in *G. hansenii *ATCC23769 in order to check the cellulose production by the genetically modified microorganism in comparison with the wild microorganism.
